# Supplementary material for: Causal Relationship between Aging and Anorexia Nervosa: A White-Matter-Microstructure-Mediated Mendelian Randomization Analysis
Source: Biomedicines. 2024 Aug 16;12(8):1874. doi: 10.3390/biomedicines12081874 (PMC11351342; doi:10.3390/biomedicines12081874)
Supplement: Supplementary file 1 [file biomedicines-12-01874-s001.zip › Supplementary Figure/Figure S4/Singe SNP plot of MR analysis for TL to ubm-b-2108.pdf]

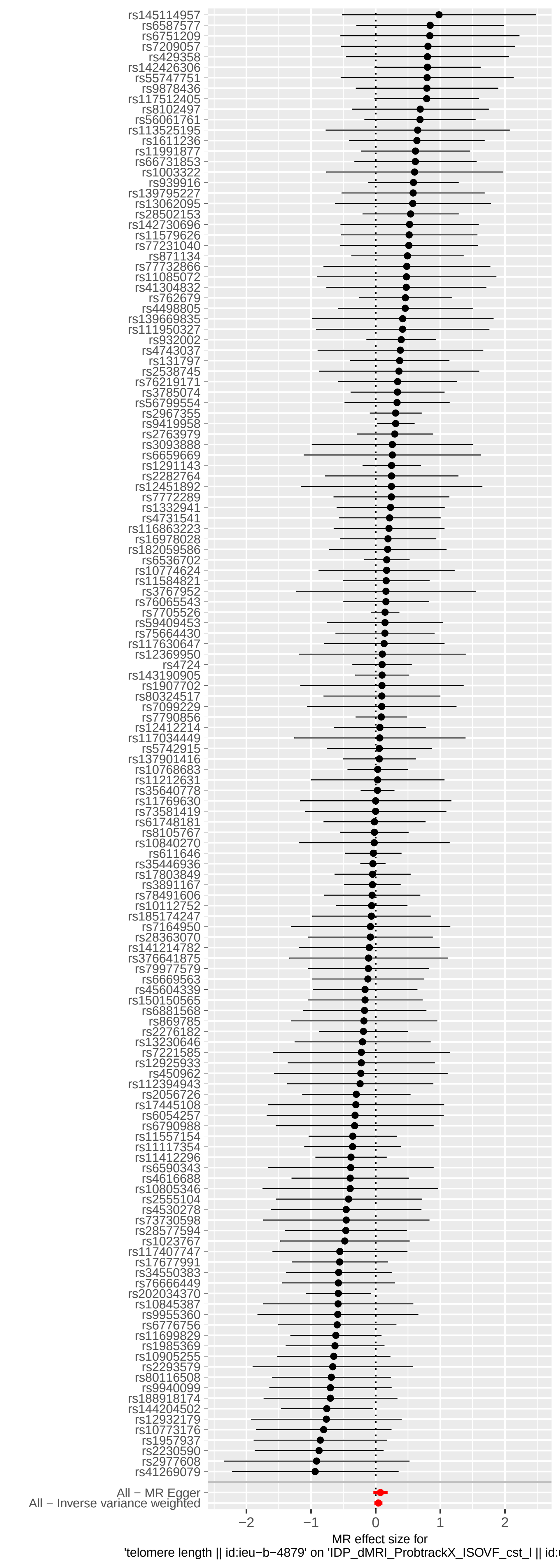
rs145114957
rs6587577
rs6751209
rs7209057
rs429358
rs142426306
rs55747751
rs9878436
rs117512405
rs8102497
rs56061761
rs113525195
rs1611236
rs11991877
rs66731853
rs1003322
rs939916
rs139795227
rs13062095
rs28502153
rs142730696
rs11579626
rs77231040
rs871134
rs77732866
rs11085072
rs41304832
rs762679
rs4498805
rs139669835
rs111950327
rs932002
rs4743037
rs131797
rs2538745
rs76219171
rs3785074
rs56799554
rs2967355
rs9419958
rs2763979
rs3093888
rs6659669
rs1291143
rs2282764
rs12451892
rs7772289
rs1332941
rs4731541
rs116863223
rs16978028
rs182059586
rs6536702
rs10774624
rs11584821
rs3767952
rs76065543
rs7705526
rs59409453
rs75664430
rs117630647
rs12369950
rs4724
rs143190905
rs1907702
rs80324517
rs7099229
rs7790856
rs12412214
rs117034449
rs5742915
rs137901416
rs10768683
rs11212631
rs35640778
rs11769630
rs73581419
rs61748181
rs8105767
rs10840270
rs611646
rs35446936
rs17803849
rs3891167
rs78491606
rs10112752
rs185174247
rs7164950
rs28363070
rs141214782
rs376641875
rs79977579
rs6669563
rs45604339
rs150150565
rs6881568
rs869785
rs2276182
rs13230646
rs7221585
rs12925933
rs450962
rs112394943
rs2056726
rs17445108
rs6054257
rs6790988
rs11557154
rs11117354
rs11412296
rs6590343
rs4616688
rs10805346
rs2555104
rs4530278
rs73730598
rs28577594
rs1023767
rs117407747
rs17677991
rs34550383
rs76666449
rs202034370
rs10845387
rs9955360
rs6776756
rs11699829
rs1985369
rs10905255
rs2293579
rs80116508
rs9940099
rs188918174
rs144204502
rs12932179
rs10773176
rs1957937
rs2230590
rs2977608
rs41269079
All − MR Egger
All − Inverse variance weighted
−2
−1
0
1
2
MR effect size for
'telomere length || id:ieu−b−4879' on 'IDP_dMRI_ProbtrackX_ISOVF_cst_l || id:i
